# 急性髓系白血病患者异基因造血干细胞移植前后监测NUP98::NSD1融合基因的临床意义

**DOI:** 10.3760/cma.j.issn.0253-2727.2023.12.007

**Published:** 2023-12

**Authors:** 亚可 尚, 信安 潘, 英军 常, 亚溱 秦, 昱 王, 晨华 闫, 于谦 孙, 晓军 黄, 晓甦 赵

**Affiliations:** 北京大学人民医院，北京大学血液病研究所，国家血液系统疾病临床医学研究中心，造血干细胞移植治疗血液病北京市重点实验室，北京 100044 Peking University People's Hospital & Peking University Institute of Hematology, National Research Center for Hematologic Disease, Beijing Key Laboratory of Hematopoietic Stem Cell Transplantation, Beijing 100044, China

**Keywords:** 急性髓系白血病, 异基因造血干细胞移植, 可检测残留病, 复发, 融合基因，NUP98::NSD1, Acute myeloid leukemia, Allogeneic hematopoietic stem cell transplantation, Measurable residual disease, Relapse, Fusion gene, NUP98::NSD1

## Abstract

**目的:**

观察急性髓系白血病（AML）患者异基因造血干细胞移植（allo-HSCT）前后核孔蛋白98（NUP98）::NSD1融合基因表达的动态变化，并初步分析其作为可检测残留病（MRD）评估指标预测移植后白血病复发的临床价值。

**方法:**

16例在北京大学人民医院诊断为NUP98::NSD1融合基因阳性AML并接受allo-HSCT的患者纳入研究，移植前后监测NUP98::NSD1融合基因、流式细胞术（FCM）检测白血病免疫残留等指标来评估其MRD状态。

**结果:**

所有患者中位随访时间为526（139～1136）d，共有4例（25.0％）患者在移植后出现血液学复发，中位复发时间为474（283～607）d。3例（18.8％）患者死亡，其中2例（12.5％）死于白血病复发。数据齐全的初诊患者在初诊时NUP98::NSD1的中位表达水平为78.550％（18.900％～184.400％）。移植后NUP98::NSD1阳性患者复发率更高，NUP98::NSD1阳性患者中44.4％发生复发，移植后NUP98::NSD1阴性患者中无发生复发。移植后NUP98::NSD1水平预测复发的ROC曲线下面积（AUC）＝1.000（95％*CI* 1.000～1.000，*P*＝0.003）。4例复发患者中，NUP98::NSD1较FCM检测免疫残留和Wilms肿瘤基因1（WT1）更为敏感。

**结论:**

NUP98::NSD1融合基因可用于评估该类AML的MRD状态。移植后NUP98::NSD1阳性患者复发率高，预后差。NUP98::NSD1 比FCM和WT1预测移植后复发更为敏感。

## Abstract (en)

**Objective:**

This study aimed to observe the dynamic changes of NUP98::NSD1 expression before and after allogeneic hematopoietic stem cell transplantation（allo-HSCT）. Moreover, the clinical value of measurable residual disease（MRD）was analyzed.

**Methods:**

Sixteen AML patients who were diagnosed with the NUP98::NSD1 fusion gene and received allo-HSCT at Peking University People's Hospital were included. The NUP98::NSD1 fusion gene and leukemia-associated immunophenotype（LAIP）were monitored before and after transplantation to evaluate their MRD status.

**Results:**

The median follow-up time for all patients was 526 days（139-1136 days）, with four patients（25.0％）experiencing hematological recurrence at a median of 474 days（283-607 days）after transplantation. Three patients（18.8％）died, two of whom（12.5％）died of leukemia recurrence. The median expression level of NUP98::NSD1 in newly diagnosed patients with complete data was 78.5％（18.9％-184.4％）at the time of initial diagnosis. The recurrence rate was higher in NUP98::NSD1-positive patients after transplantation, with 44.4％ of patients experiencing recurrence, whereas no recurrence occurred in NUP98::NSD1-negative patients after transplantation. The area under the receiver operating characteristic curve predicted by the NUP98::NSD1 level after transplantation was 1.000（95％ confidence interval: 1.000-1.000, *P*＝0.003）. Among the four patients with recurrence, NUP98::NSD1 was more sensitive than flow cytometry residual（FCM）and Wilms' tumor gene 1（WT1）.

**Conclusion:**

The NUP98::NSD1 fusion gene can be used to evaluate the MRD status of allo-HSCT. NUP98::NSD1-positive patients after transplantation have a high relapse rate and poor prognosis. NUP98::NSD1 was more sensitive than FCM and WT1 in predicting posttransplant relapse.

既往研究表明，遗传标志对恶性肿瘤的诊断和预后具有重要意义[1]–[2]。而可检测残留病（MRD）是预警急性白血病患者复发风险的重要指标，以MRD为指导的干预可降低复发率[3]–[4]。对于伴有白血病特异性融合基因的患者，采用实时定量聚合酶链反应（RQ-PCR）定量检测这些融合基因可以准确、敏感地反映MRD状态。NUP98::NSD1是一种罕见的髓系肿瘤相关融合基因，由涉及染色体11p15上的NUP98基因的染色体易位产生，是急性髓系白血病（AML）复发和难治性儿童疾病中常见的基因型，预后较差[5]–[7]。allo-HSCT可显著改善NUP9 重排AML患者的预后[8]。目前临床研究分析NUP98::NSD1融合基因在监测MRD状态和预测allo-HSCT后复发的临床意义较少。本研究中，我们回顾性分析了在本中心接受allo-HSCT的NUP98::NSD1阳性AML患者的连续队列，在移植后特定时间点用RQ-PCR法监测NUP98::NSD1融合基因表达变化，并比较了NUP98::NSD1表达水平与其他MRD标志物预警白血病复发的效能，分析NUP98::NSD1是否适合作为预警移植后复发的MRD标志。

## 病例与方法

一、研究对象

本研究纳入2019年9月至2022年9月在北京大学血液病研究所接受allo-HSCT的16例NUP98::NSD1阳性AML患者，使用世界卫生组织（WHO）标准[9]对患者进行诊断。本研究得到北京大学人民医院伦理委员会批准（批件号：2019PHB15801）。全相合同胞供者造血干细胞移植（MSD-HSCT）1例，单倍体造血干细胞移植（haplo-HSCT）15例。

二、移植方案

本研究中纳入的患者接受清髓预处理。haplo-HSCT：阿糖胞苷（Ara-C）4 g·m^−2^·d^−1^静脉滴注，−10 d、−9 d；白消安（BU）3.2 mg·kg^−1^·d^−1^静脉滴注，−8 d～−6 d；环磷酰胺（CTX）1.8 g·m^−2^·d^−1^，−5 d、−4 d；司莫司汀（Me-CCNU）250 mg/m^2^，−3 d；兔抗人胸腺细胞免疫球蛋白（rATG）2.5 mg·kg^−1^·d^−1^，−5 d～−2 d。MSD-HSCT：白消安、CTX、司莫司汀用法同上，加用羟基脲（总量80 mg/kg），Ara-C单日剂量减半，不用rATG。所有患者均接受经过粒细胞集落刺激因子（G-CSF，5 µg·kg^−1^·d^−1^，连续5 d）刺激的外周血干细胞和骨髓输注。所有患者均以环孢素A+霉酚酸酯+短程甲氨蝶呤预防GVHD。

三、WT1与NUP98::NSD1基因分析

采用基于TaqMan的RQ-PCR方法检测NUP98::NSD1融合基因。所有病例均采用骨髓样本进行RQ-PCR检测，内参基因为ABL。NUP98::NSD1融合基因表达水平（％）＝NUP98::NSD1融合基因拷贝数/ABL基因拷贝数×100％，NUP98::NSD1转录水平>0定义为阳性。WT1基因表达水平（％）＝WT1基因拷贝数/ABL基因拷贝数×100％，WT1转录水平>0.6％定义为阳性。

四、定义

主要研究终点为白血病复发，次要终点为死亡。生存时间为从移植后开始到最后随访时间或终点事件发生时间。参照文献[10]方法采用多色流式细胞术（FCM）进行MRD检测，FCM阳性定义为FCM检测骨髓异常髓系幼稚细胞≥0.01％。移植前测量是指移植前1个月内采集的骨髓样本，移植后采样时间点为移植后1、2、3、4.5、6、9、12个月，此后根据患者个人意愿，对部分患者进行了更频繁的MRD监测。移植后MRD阳性患者的干预措施包括化疗、干扰素、减停免疫抑制剂和（或）供者淋巴细胞输注（DLI）等。

五、统计学处理

使用SPSS 26.0进行统计分析。采用受试者工作特征（ROC）进行诊断分析。双侧*P*值小于0.05被认为有统计学意义。

## 结果

一、患者特征

本研究纳入16例NUP98::NSD1阳性AML患者，其中男10例，女6例，中位年龄24（5～60）岁。中位随访时间为526（139～1136）d。共有4例（25.0％）患者在移植后出现血液学复发，中位复发时间为移植后474（283～607）d。3例（18.8％）患者死亡，其中2例（12.5％）死于白血病复发。16例患者中6例缺失初诊NUP98::NSD1检查结果，其余10例患者的NUP98::NSD1中位表达水平为78.550％（18.900％～184.400％）。入组患者的临床资料与检测结果见表1。

**表1:** 16例NUP98::NSD1融合基因阳性急性髓系白血病（AML）患者临床资料

例号	年龄（岁）	性别	FAB分型	移植前WT1（%）	初诊NUP98::NSD1（%）	移植前NUP98::NSD1（%）	复发前NUP98::NSD1峰值（%）	移植前状态	移植资料	随访结局	随访时间（d）
1	26	女	M_5_	0	100	100	0.039	NR	其父HLA5/10相合，B+供B+	无事件生存	255
2	36	男	M_4_	9.900	37.000	120.200	0.330	CR，MRD+	母HLA3/6相合，B+供B+	复发	354
3	33	女	M_4_	6.700	66.500	11.900	0.015	CR，MRD+	其子HLA5/10相合，O+供A+	无事件生存	372
4	59	女	M_5_	21.200	/	137.000	0.014	CR，MRD+	女儿HLA5/10相合，A+供A+	治疗相关死亡	353
5	5	女	M_4_	0.280	55.000	0.220	0	CR，MRD-	兄HLA10/10相合，A+供A+	无事件生存	95
6	54	男	M_5_	0.530	/	0.016	0	CR，MRD+	子供父	无事件生存	219
7	6	男	M_2_	4.100	/	5.626	0.009	CR，MRD+	母亲HLA 5/10相合，B+供B+	无事件生存	365
8	22	男	M_4_	3.000	48.800	48.800	0.025	CR，MRD+	父亲HLA5/10相合，B+供B+	无事件生存	98
9	6	男	M_4_	0.068	18.900	0	0.380	CR	父供子，HLA 4/6，A+供O+	复发	332
10	46	男	M_2_	11.800	184.400	13.900	17.600	NR	女供父，HLA5/10，B+供O+	复发	95
11	60	男	M_4_	162.700	90.600	0	0.570	NR	子供父，HLA3/6，O+供O+；女供父HLA3/6，O+供O+	复发	292
12	27	女	M_2_	0.330	99.300	0.055	0	CR	父供女，HLA5/10，AB+供A+	无事件生存	207
13	7	男	M_2_	0.300	/	0.830	0.014	CR	父供子 HLA3/6 AB+供AB+	无事件生存	86
14	18	男	M_2_	0.240	/	4.400	0	CR	父供子，HLA5/10 B+供B+	无事件生存	248
15	6	男	M_2_	0	/	0	0	CR	父供子HLA5/10B+供AB	无事件生存	43
16	7	女	M_5_	1.700	370.100	24.000	0	CR	父供女 HLA5/10 A+供A+	无事件生存	114

注 NR：未缓解；CR：完全缓解；MRD：可检测残留病。/：数据缺失

二、移植前NUP98::NSD1融合基因表达

16例患者中，3例移植前NUP98::NSD1阴性，13例患者移植前NUP98::NSD1阳性，移植前NUP98::NSD1中位表达水平为12.900％（0～120.200％）。这13例移植前NUP98::NSD1阳性患者中有8例移植后出现NUP98::NSD1阳性，其中6例患者在移植后早期（3个月内）出现NUP98::NSD1阳性，其中5例NUP98::NSD1表达水平较低，中位数为0.008％（0.006％～0.014％），之后在干扰素干预后转阴，而另1例患者在移植后1个月NUP98::NSD1表达水平为17.600％，最终干预无效发生复发。8例移植前、后NUP98::NSD1均为阳性的患者中，1例在移植后12个月出现首次移植后NUP98::NSD1阳性（表达量0.025％），3例患者在移植后250（180～300）d检出NUP98::NSD1阳性，干预无效发生血液学复发。而3例移植前NUP98::NSD1阴性患者中只有1例在移植后出现NUP98::NSD1阳性（在NUP98::NSD1达到0.380％后发生血液学复发）。对移植前检测到的NUP98::NSD1水平进行ROC曲线分析。结果显示曲线下面积为0.417（95％*CI* 0.018～0.826，*P*＝0.628），提示移植前NUP98::NSD1阳性不能作为移植后复发的有效预测指标。

三、移植后NUP98::NSD1融合基因表达

共有10例（61.1％）患者在移植后NUP98::NSD1融合基因再次阳性，中位检出时间为移植后90（30～270）d。复发前NUP98::NSD1峰值中位表达水平为0.196％（0.009％～17.600％）。10例患者中有4例在随访期间发生白血病复发，4例复发患者移植后NUP98::NSD1均为阳性。复发与死亡只发生于移植后NUP98::NSD1阳性组中，提示移植后NUP98::NSD1阳性可预警复发。为进一步分析NUP98::NSD1在预测复发中的价值，对移植后复发前检测到的NUP98::NSD1最高表达水平进行ROC曲线分析。结果显示AUC＝1.000（95％*CI* 1.000～1.000，*P*＝0.003）（图1），说明NUP98::NSD1阳性在移植后可以作为有效的复发预测指标，该融合基因预测复发的最佳界值为0.200％。移植后10例NUP98::NSD1阳性患者中4例复发患者的NUP98::NSD1表达水平均高于0.200％，6例非复发患者中NUP98::NSD1表达均<0.200％。

**图1:**
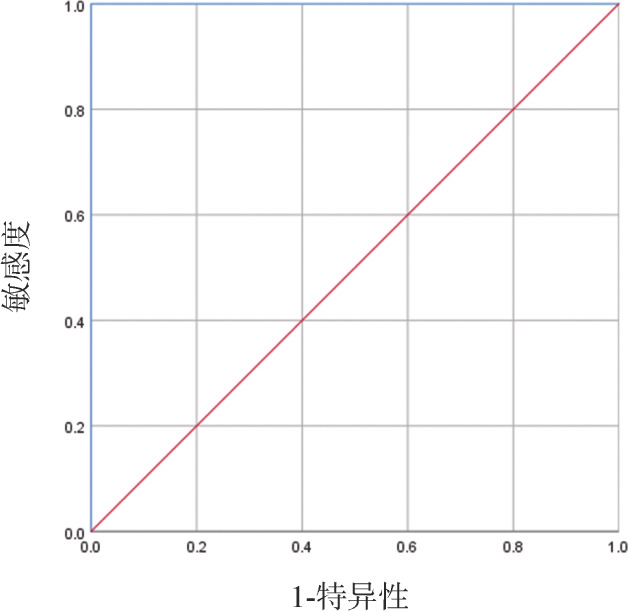
移植后NUP98::NSD1阳性预测复发的受试者工作特征曲线

四、移植后NUP98::NSD1表达与WT1以及FCM对比

我们对比了NUP98::NSD1阳性以及WT1表达两者作为移植后复发指标的效能。16例NUP98::NSD1基因表达患者移植后通过NUP98::NSD1表达，WT1表达和FCM检测MRD状态见图2。MRD阴性定义为NUP98::NSD1、WT1和FCM检测在任何时间点都为阴性。结果显示，4例移植后复发患者（例2、9、10、11）在复发前NUP98::NSD1阳性（图2），其中3例患者（例9、10、11）NUP98::NSD1阳性发生于FCM阳性之前（图2）。3例移植后复发患者（例2、11、13）复发前NUP98::NSD1阳性都早于WT1表达（图2），1例移植后复发患者（例12）在移植后1个月NUP98::NSD1与WT1均为阳性（图2）。上述结果提示NUP98::NSD1可以比FCM和WT1更早预测移植后复发。

**图2:**
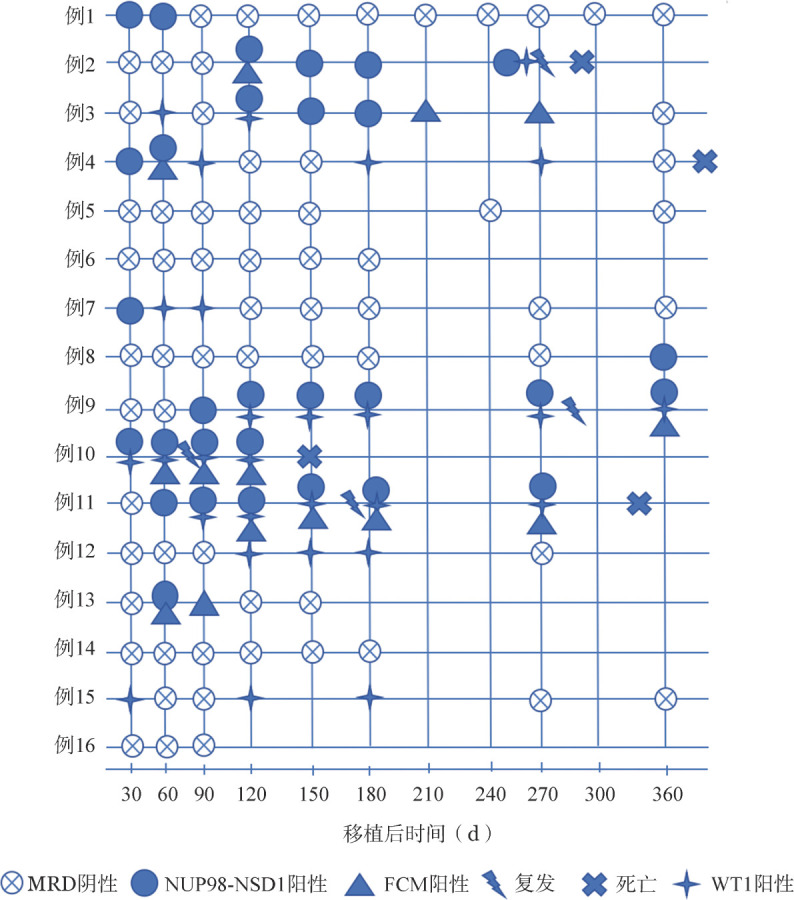
16例NUP98::NSD1融合基因阳性急性髓系白血病（AML）患者异基因造血干细胞移植后NUP98::NSD1、WT1表达和流式细胞术（FCM）检测结果

为进一步比较WT1以及NUP98::NSD1在复发患者中的监测复发的效能，将4例复发患者移植后1、2、3、4.5、6、9、12个月的NUP98::NSD1融合基因和WT1基因表达量进行分析。例2于移植后9个月复发，复发前未检出WT1阳性，从移植后4个月开始检出NUP98::NSD1阳性。例9于移植后10个月复发。在复发之前WT1在移植后6、9个月检测到2次阳性，而NUP98::NSD1在移植后3、4.5、9个月均为阳性表达呈上升趋势。例10于移植后2个月复发，WT1在移植后1个月检出阳性，呈轻微上升趋势；移植后1个月NUP98::NSD1表达水平呈高度上升趋势。例11于移植后4个月复发，移植后3个月检出WT1阳性；NUP98::NSD1在移植后2、3个月均为阳性且呈上升趋势。综上所述，NUP98::NSD1相比于WT1和FCM更能提前和精准地监测移植后患者复发。

五、移植后复发患者的干预与预后

我们分析了11例MRD阳性患者在移植后的干预效果。MRD阳性时干预措施主要包括干扰素注射、化疗联合DLI。4例复发患者在MRD阳性时给予干扰素、化疗联合DLI干预，但最终均复发。另外7例患者接受化疗联合DLI，MRD转阴，NUP98::NSD1融合基因阴性。

## 讨论

NUP98::NSD1是一种罕见的白血病融合基因。由于NUP98::NSD1阳性AML患者数量有限，目前尚无报道关注NUP98::NSD1表达对移植后白血病复发的预测意义。在我们的研究中，我们观察分析了18例NUP98::NSD1阳性并接受移植的AML患者，结果表明NUP98::NSD1融合基因在移植后MRD监测中预测复发具有意义，同时预测复发的效果优于WT1和FCM。

大多数研究表明，移植前MRD阳性预示移植后的高复发风险[11]–[13]。然而，我们的研究表明移植前NUP98::NSD1表达阳性不能作为复发的预测指标，这可能由于NUP98::NSD1定量检测非常敏感，移植前低水平的NUP98::NSD1融合基因并不一定意味着移植后的复发风险，极低水平的NUP98::NSD1可能被预处理清除，也可能在移植后移植物抗白血病（GVL）的作用下被供者免疫细胞清除。本研究样本量较小，需要大样本研究来确认。

本研究结果初步显示NUP98::NSD1融合基因可能是一个特异、敏感的MRD生物标志物。在本研究中，复发全部发生于移植后NUP98::NSD1阳性组，可见NUP98::NSD1在移植后表达阳性对复发有着特异的预警意义。NUP98::NSD1融合基因在初诊时高表达，移植前逐渐转变为阴性或低表达。在移植后复发前再次呈阳性。这种动态变化与临床表现、肿瘤负荷及流式残留的水平基本一致。此外，研究结果显示，NUP98::NSD1表达先于FCM阳性和血液学复发，提示NUP98::NSD1的分子检测可能比FCM更敏感。考虑到NUP98::NSD1基因表达的动态变化及肿瘤倍增时间，移植后早期需每月进行骨髓监测。

既往研究表明，无GVHD的allo-HSCT后复发患者停用免疫抑制剂后DLI可诱导缓解[14]。Yan等[14]研究显示，MRD阳性患者接受化疗后再行DLI，3年累积复发率（CIR）、无病生存（DFS）率、OS率分别为26.4％、51.2％、60.5％。本研究中有7例患者在NUP98::NSD1阳性后使用DLI等手段进行干预获得良好的疗效，他们在进行干预时的NUP98::NSD1中位表达水平为0.015％（0.009％～0.062％），而复发的4例患者在进行干预时的NUP98::NSD1中位表达水平为0.196％（0.009％～17.600％），两组的中位表达水平存在较大差异。这一结果提示对于NUP98::NSD1阳性AML患者，可能在移植后NUP98::NSD1水平较低时进行干预效果较好。NUP98::NSD1高表达的患者还需探索新的复发治疗策略。

综上所述，本研究结果提示NUP98::NSD1融合基因可作为AML患者allo-HSCT后的MRD指标，NUP98::NSD1融合基因预测复发可能性可能优于FCM、WT1。基于NUP98::NSD1表达的早期复发干预可能进一步降低复发率，提高移植疗效。同时本研究中由于NUP98::NSD1融合基因AML的患者数量有限，其表达水平在移植前后的动态变化及意义还需要继续探索。
